# Perceived policy-related barriers to palliative care implementation: a qualitative descriptive study

**DOI:** 10.1177/26323524231198542

**Published:** 2023-09-11

**Authors:** Atsede Aregay, Margaret O’Connor, Jill Stow, Nicola Ayers, Susan Lee

**Affiliations:** Health and Nursing Sciences, University of Agder, Høvågveien 140, Kristiansand, 4604, Norway; School of Nursing, Mekelle University, Tigray, Ethiopia; Nursing and Midwifery, Monash University, Melbourne City Mission Palliative Care, Melbourne, Australia; St Vincent’s Private Hospital, Melbourne, Australia; Nurse Lecturer, School of Nursing, BPP University, London, UK; Nursing and Midwifery, Monash University, Melbourne, Australia

**Keywords:** Palliative care, policy, healthcare, systems integration, barriers

## Abstract

**Background:**

Ethiopia has a national palliative care guideline, and palliative care is explicitly recognised in the country’s healthcare policy and health sector transformation plans. However, palliative care is not fully delivered in the regional public hospitals and primary health care units.

**Objective:**

This study explores perceived policy barriers to deliver palliative care services in rural and regional healthcare settings, which primary healthcare units largely serve.

**Design:**

Face-to-face interviews were conducted in a rural region of Ethiopia.

**Methods:**

Forty-two participants were recruited from a variety of health settings including primary, secondary and tertiary levels across the region. Interviews were conducted with policymakers from the regional health bureau, pharmacists, medical doctors, health officers (clinical officers) and nurses, including chief nursing officers in leadership roles at all levels of healthcare institutions. Data analysed using thematic analysis.

**Results:**

Participants described several barriers related to healthcare policy, including lack of government priority and focus on palliative care; lack of health professionals’ awareness of the national palliative care plans and guidelines; and lack of palliative care integration into the existing healthcare system and the national budget. Participants remarked that palliative care services in the region were mainly limited to HIV patients, often managed with external support and, hence unsustainable.

**Conclusions:**

Policy priority and focus is a fundamental component for the provision of palliative care because lack of focus and support from the government have led to inadequate access to palliative care for all in need. Hence, as participants suggested, palliative care should be integrated into all healthcare levels, particularly into the primary health care units and the health extension programme, to facilitate health extension workers to support millions living in rural areas.

## Introduction

Palliative care is a medical and ethical necessity and not an alternative form of care. Hence, it should be made available at all levels of healthcare systems and accessible to all in need of the service.^1,2^ Despite palliative care being considered a key component of universal health coverage, globally, only an estimated 12% of those in need receive it.^1,3^ In 2014, the Sixty-seventh World Health Assembly (WHA67.19) resolved that ‘palliative care is an ethical responsibility of health systems, and it is an ethical duty of healthcare professionals to alleviate pain and suffering, whether physical, psychosocial or spiritual’.^4^

As a roadmap to achieving the global aspiration for universal access to palliative care, WHO recommends a public health strategy, which includes policy formulation, promotion and improving availability and accessibility of palliative medicines to strengthen palliative care in the public healthcare system.^1,5^ Specifically, it recommends the integration of palliative care into national health policies or strategic plans on cancer, non-communicable diseases, or infectious diseases such as HIV/AIDs and drug-resistant tuberculosis.^1,6^

Globally, the integration of palliative care into the mainstream health service provision and national health policies has been recognised as an essential foundation for expanding palliative care and improving access to all in need.^1,5,7^ A review of five African Countries indicated that palliative care is well incorporated into national health policies and relevant guidelines.^7–9^ Ethiopia is ranked as the second most advanced country in Africa in palliative care policy development, mainly because it is incorporated into the country’s health policies and guidelines.^10,11^ Thus Ethiopian policy approach is consistent with the 2014 resolution of the World Health Assembly that suggested palliative integration into all national health policies and guidelines.^1,4^

In Ethiopia, palliative care is incorporated into the country’s national health sector transformation plan as a fifth health pillar, alongside primary health promotion, prevention, curative, and rehabilitation.^12^ Palliative care is also included in the national cancer control plan^13^; Ethiopian primary health care clinical guidelines^14^; Ethiopia hospital transformation guidelines^15^ as well as the national strategic action plan for prevention & control of non-communicable diseases.^16^ Ethiopia also developed a national palliative care guideline and a national target to provide palliative care in at least 50% of public health facilities by 2020.^13^ However, despite these targets and the existence of national plans and guidelines on palliative care, the healthcare professionals were not aware of these documents.^17^ In addition, there are limited government programmes or public health care facilities and small number of non-governmental hospice/palliative care institutions that deliver the service mostly found in the capital city.^17,18^ This study aims to identify perceived policy barriers to deliver palliative care services in rural and regional healthcare settings, which primary healthcare units largely serve.

## Methods

### Study design

Perceived policy barriers to delivering palliative care services were explored using a qualitative descriptive study and a pragmatist worldview approach.^19^

### Study settings

Face-to-face in-depth interviews were conducted with 42 participants recruited purposely from the northern region in Ethiopia.^20^ Like in the rest of the country, the study region provides health services to its population through a three-tiered healthcare system: namely, primary health care unit, secondary care (general hospital) and tertiary care (comprehensive specialised hospital).^12^ Interviews were conducted with policymakers from the regional health bureau, pharmacists, medical doctors, health officers (clinical officers), and nurses, including chief nursing officers in leadership roles at all levels of healthcare institutions. Additionally, academics from the university and regional health college and health extension workers deployed in health posts located in rural areas were also interviewed. Of the 42 interview participants, four were randomly excluded due to duplicated informants. Two nurses’ interviews were not included in the analysis, because in the Comprehensive Specialised Hospital, the interviewees were duplicate informants (medical wards A and B and surgical wards A and B), and the responses were similar. In addition, one community health workers and one nurse from Primary Hospital were also excluded because they were similar informants and similar responses. In total, 38 interview transcripts were analysed.

### Data collection

The qualitative study was conducted between October 2018 and January 2019 and targeted professionals who had worked at least 6 months prior to the qualitative interview in the selected institutions. Interview guides were prepared following the four components of public health strategy, focusing on policy.^5^ The interviews were conducted in the local language and facilitated by one author (AA) using an audio recorder.

### Data analysis

Data analysis was supported by the software package NVivo 12 and guided by a thematic analysis technique.^21,22^ The thematic analysis utilised the following six steps: becoming familiar with the data set by transcribing, reading and re-reading and taking notes from the data; coding core features from the data; searching and gathering the relevant data and generating initial themes; Developing potential themes and review; checking themes for relevance with the codes defining themes and writing the scholarly report of the final analysis. The emerging themes were synthesised to comprehensively identify the barriers to implementing the national policy in rural and regional healthcare settings. Translation/transcription from the recordings were undertaken by AA and de-identified to maintain confidentiality. All methods of the study were performed in accordance with the standards of the Declaration of Helsinki.^32^

## Results

Thirty-eight interviews were analysed. Three perceived barriers were identified as follows: perceptions about prioritising and focusing on palliative care; awareness and availability of national palliative care policies, guidelines and related documents; and integration of palliative care into the existing healthcare system and the national budget. The summaries of each of these barriers are now described, drawing on interview data.

### Perceptions about the priority and focus on palliative care

Participants noted that despite increasing trends in life expectancy and the incidence of non-communicable diseases, the government’s attention and the priorities of healthcare organisations towards palliative care had not changed. A general hospital doctor remarked that: ‘*. . . the focus . . . from the federal ministry of health . . . to the regional health bureau . . . and administrative levels . . . is still on curative (in hospital* [*settings*]*) and prevention (in health centres) . . . they did not give focus on palliative care*’ (Participant 1GHD). However, some participants noted the presence of some form of palliative care service in hospitals, but only for patients with HIV. For example, a general hospital’s nurse leader confirmed that: ‘*. . .palliative care and rehabilitation are unavailable . . .*[for] *those suffering from non-communicable conditions disease, except for HIV patients.*’ (Participant 2GHND). Another primary hospital leader added: ‘*The policy . . . focus is on prevention, nutrition, family planning and delivery for mothers. . . .*’ (Participant 1PHHO).

The focus of care on maternal and child health rather than palliative care, was justified as being not a priority: ‘*Palliative care is a sort of luxury . . . our focus now should be on maternal and child health . . . There are many things that need to be prioritised in this place. . .*’ (Participant 2GHD). A university participant reflected on other health priorities, particularly in relation to non-communicable diseases and basic healthcare: ‘*. . . There are many programs such as ANC* [Antenatal care], [*safe*] *delivery, common communicable diseases, including* [Childhood] *illnesses and specialist areas that need prioritisation. . . So, I am not saying palliative care is not important, it is important . . . I will put it as second or third line. . . .*’ (Participant CSH2). ‘*Of course, we are aware of the increasing burden of non-communicable diseases* [however] *. . . but first, we must provide basic health care. In Ethiopia, most of the population* [live in rural areas]; they *are farmers* [and have] *nobody cares for them. They do not have clean water to drink, so palliative care is a luxury . . .*’ the same participant added.

Further consequences of palliative care being a low priority were described: ‘*. . .health professionals are motivated to implement the government directions . . . Even the community are following the* [footsteps] *of top leaders’ direction . . .*[However], *the government did not give priority to it* [palliative care] *. . .*’ (Participant 2PHND). Health extension workers described the lack of government support as the reason why they failed to give attention to palliative care: ‘*. . . the government did not give priority to palliative care . . . we have many activities to implement. We are focusing on high burden type of diseases* [communicable disease] *. . .*’ (Participant 1HEWHP).

Others attributed inadequate government attention to palliative care to competing needs requiring more focus: ‘*the government . . . framed its actions and funding modalities around areas of major concern. So, in the . . . past, communicable disease, low vaccination rates, . . . access to antenatal care, and . . . childbirth were key priorities for the country . . . . palliative care is not neglected by the government. The issue is about priority.*’ (Participant CRHBR). The same participant clarified that palliative care is gaining traction in the region and the country because of its applicability to care of those with non-communicable diseases: ‘*. . . our country or our region is undergoing an epidemiological transition, from communicable disease to non-communicable disease . . . So, our current priorities are informed by these transitions . . . For example, palliative care for cancer has now been accorded the required emphasis in the five years plan . . . .*’ (Participant CRHBR). Another participant added that ‘*. . . when you see the population pyramid . . . our life expectancy increases. Non communicable diseases are also increasing. So, we have obvious reasons to say that palliative care* [should be] *a priority . . .*’ (Participant CNGOR).

### Awareness of and access to national policies, guidelines and related documents on palliative care

Awareness of national health policies and guidelines on palliative care was limited to those in leadership roles and yet to filter down to nurses and clinical officers working in health facilities, or academic leaders training the future workforce in educational institutes. It was overwhelmingly reported that no palliative care documents were available at the health centre or the health post. Similarly, participants in the general hospitals and primary health care units indicated that there were no national palliative care policies and guidelines in their region, only a few documents that included palliative care. They noted a pain management guideline, the Ethiopia hospital service transformation guideline, and WHO Module in PowerPoint form, and even these were only available for the leadership of the general and primary hospitals. The comprehensive specialised hospital medical and nurse leaders reported that: ‘*. . . we had the materials like a* [national palliative care] *guideline.* [However] *the guideline is* [only] *available with the palliative care committee*’ (Participant CTHND). This was confirmed by participants from the educational college and the comprehensive specialised hospital clinical ward leaders that: ‘*. . . if there is a national guideline, we must know it, but this is the problem. . .we do not know about these documents* [palliative care] *. . .*’ (Participant CRCR).

A comprehensive specialised hospital nurse commented, ‘*. . . no policy or plan has so far come to our ward . . . we do not have a policy document related to palliative care . . .*’ (Participant CTHWNH3). A general hospital doctor also reported that palliative care policy documents were unavailable in the hospitals ‘*. . . to talk about the status of palliative care, it* [palliative care policy] *should be available in the first place . . .* [However] *palliative care is not* [implemented] *as a protocol or policy*’ (Participant 2GHD). Another primary health care unit’s participant added, ‘*. . . there is no policy designed for palliative care . . . we are not implementing palliative care in our institution. . . .*’ (Participant 1HCHO).

### Integration of palliative care into the existing healthcare system and the national budget

Participants observed that palliative care needs to be integrated as part of mainstream health programmes and allocated a dedicated budget. A regional health bureau participant noted that: ‘*. . . I do not think palliative care is integrated into the existing health system. . .*’ (Participant CNGOR). A health extension workers confirmed that palliative care is not incorporated into their activities: ‘*our work was. . . on communicable diseases, but now . . . there are 18 components, incorporating non-communicable diseases. But still, we do not have a government direction to provide palliative care* . . .’ (Participant 1HEWHP). A comprehensive specialised hospital participant also suggested to start integration from the community: ‘*. . . integration is a major problem . . . but the hospital system is not an island. It* [Palliative care] *should be integrated* [throughout the system including at], *the community* [level]*. . . .*’ (Participant CTHD).

However, participants suggested that just as other specialties were recently integrated into the existing health extension programme, so could palliative care. For example: ‘*The first health extension workers had 16 components; now we add two programs, non-communicable diseases, and mental illness . . . so, we already have the system, the thing is . . . we need to integrate . . . those services* [palliative care] *into the system starting from health posts, to . . . comprehensive specialised hospital* [network] *. . . we have a standard for each level* [and] *. . . our community is aware of these . . .’* (Participant CRHBR). Comprehensive specialised hospital and general hospital participants suggested budget allocation for palliative care ‘*. . . we are using from the available budget.* [However], *when* [palliative care and other] *initiatives are launched by the ministry of health, there should be a discussion with the ministry of finance and economic development to ensure that they have a dedicated budget to implement the initiatives. . .*’ (Participant CTHD).

## Discussion

The study findings describe the perceived policy-related barriers to deliver palliative care services in rural and regional healthcare settings. The key findings indicate that the government provide low priority and focus to palliative care services. Participants also described the inconsistent practice because palliative care is not integrated into the existing healthcare system and into the national budget. Although palliative care included into different national healthcare plans and guideline, participants indicated that the healthcare workers have lack of awareness about and access to the palliative care documents excepts a few leaders. All study participants said that health professionals serving in clinical wards and educational leaders training the future workforce of the country needed access to information about both policy and guidelines. Participants remarked that only a few leaders from the healthcare institutes were familiar with these documents and had access to them. Thus, there is a significant gap between policy and guideline development, and their access and applicability for clinicians. Participants argued that health professionals need to be made aware of the presence of palliative care in the national policy, similar to the study conducted in Ethiopia by Kaba *et al*.^17^

In 2014, the World Health Assembly recommended the integration of ‘evidence-based, cost-effective, and equitable palliative care services in the continuum of care, across all levels of health service, emphasising primary care, community- and home-based care, and universal coverage schemes’.^4^ In addition, the WHO public health strategy, which was used to frame this study, recommended that the government integrate palliative care into all levels of the healthcare system, and palliative care should be owned by the community.^5,17,18,23^ In Ethiopia, there are a few palliative care services located in Addis Ababa, the capital city, and a few non-governmental organisations such as Hospice Ethiopia providing home-based palliative care, but all are donor-dependent.^17,18,24^ Further, these services have not spread into rural and regional healthcare settings where millions of people reside.

A large multi-country palliative care survey conducted in 2020^25^ categorised Ethiopia as a country which is limited in population coverage and is donor dependent, making the service unsustainable. That this study revealed palliative care as not well integrated into the mainstream health care system is in alignment with the previous study. In another study,^26^ it was found that lack of care integration within existing healthcare systems represents the main challenge to palliative care access and provision for low- and middle-income countries.

Participants suggested that palliative care could be integrated into public hospitals and primary health care units, particularly into the health extension programme. The WHO also recommended integrating palliative care into primary healthcare and described the role of community healthcare workers in palliative care, such as providing emotional support for patients and families.^2^ In this study, health extension workers deliver care for those with non-communicable diseases, and mental health conditions, and are part of what is offered to the community. Incorporating palliative care as part of one of the roles of community healthcare workers or health extension workers may be cost-effective and improve access, especially in hard-to-reach communities. This is similar to the experience in rural South Africa, where community healthcare workers contribute to expanding palliative care access to patients, guided and supervised by nurses.^27^

Participants stated that introducing and maintaining integrated palliative care in healthcare settings required a dedicated budget to ensure that the services were sustainable and delivered to all in need. The qualitative findings revealed this has not been so in the study area, where even if palliative care had been offered in a limited setting (such as that implemented for those with HIV), they are largely dependent on donors for financial support. These findings are consistent with previous studies conducted in low- and middle-income countries where palliative care initiatives commenced with a project supported by third-party donors and grants^8,28,29^ and this has led many participants to raise sustainability as a significant service delivery issue. However, evidence from Uganda suggests that integrating palliative care into the healthcare system, and embedding the activities and costs in the national healthcare budget is an essential first step to ensuring sustainability and meeting growing community needs.^7^ Accordingly, for Ethiopia to achieve the national goal of expanding palliative care to 50% of healthcare facilities, there is a need to prioritise palliative care and ensure the programme is fully funded.^13^

## Limitations

This study was deliberately conducted in one of the eleven regions in Ethiopia and therefore findings may not be generalisable to the rest of the Country. Hence, the study findings should be applied considering the different regional contexts of the Country. In addition, there may be changes in the focus and priority of the national health policies and guidelines. The previously available health service may not provide routine healthcare due to the COVID-19 pandemic and the recent civil war in Ethiopia.

## Afterword

This study was undertaken before the recent civil war in Ethiopia (from November 2020 to 2022), when health care systems were functional. The war caused major disruption to all aspects of life, with most health systems in the conflict region unable to function normally. Others have been either deliberately attacked or looted, resulting in limited services and care staff.^30,31^ It is hoped that recent cease-fire efforts will facilitate a return to normal life.

## Conclusion

This study explored the perceived barriers to integrating palliative care into Ethiopia’s rural and regional health care settings. While palliative care is included in different national healthcare plans and guidelines, these documents need to be more effectively distributed throughout all levels of the healthcare system. Palliative care needs to be prioritised, more effectively integrated into the existing healthcare system and funded through the national health budget. As participants suggested, palliative care should be integrated into the existing healthcare system and particularly into the activities of health extension workers, akin to models of care for those with non-communicable diseases and mental health.

## Declarations

**Ethics approval and consent to participate:** Ethics approval for the study was gained from an Ethiopian university Ethical Review Committee ERCs (1494) and HREC, Monash University, Australia (13402). Interview participants responded to circulated explanatory statements and posters and contacted the researcher AA directly to arrange interviews. Interviewees provided written informed consent before their interview. All transcripts were de-identified before analysis, and the interviewees were identified only by role and employer type.

**Consent for publication:** Not applicable.

**Availability of data and materials:** The datasets used and/or analysed during the current study are available from the corresponding author on reasonable request.
